# Ion transport its regulation in the endolymphatic sac: suggestions for clinical aspects of Meniere’s disease

**DOI:** 10.1007/s00405-016-4362-1

**Published:** 2016-11-01

**Authors:** Nozomu Mori, Takenori Miyashita, Ryuhei Inamoto, Ai Matsubara, Terushige Mori, Kosuke Akiyama, Hiroshi Hoshikawa

**Affiliations:** 1 Osaka Bay Central Hospital, 1-8-30 Chikkou, Minato-ku, Osaka, 552-0021 Japan; 2 Department of Otolaryngology, Faculty of Medicine, Kagawa University, Kita-gun, Miki-cho, Ikenobe 1750-1, Kagawa, 761-0793 Japan

**Keywords:** Endolymphatic sac, Mitochondria-rich cells, Sodium ion transport, Aldosterone

## Abstract

Ion transport and its regulation in the endolymphatic sac (ES) are reviewed on the basis of recent lines of evidence. The morphological and physiological findings demonstrate that epithelial cells in the intermediate portion of the ES are more functional in ion transport than those in the other portions. Several ion channels, ion transporters, ion exchangers, and so on have been reported to be present in epithelial cells of ES intermediate portion. An imaging study has shown that mitochondria-rich cells in the ES intermediate portion have a higher activity of Na^+^, K^+^-ATPase and a higher Na^+^ permeability than other type of cells, implying that molecules related to Na^+^ transport, such as epithelial sodium channel (ENaC), Na^+^–K^+^–2Cl^−^ cotransporter 2 (NKCC2) and thiazide-sensitive Na^+^–Cl^−^ cotransporter (NCC), may be present in mitochondria-rich cells. Accumulated lines of evidence suggests that Na^+^ transport is most important in the ES, and that mitochondria-rich cells play crucial roles in Na^+^ transport in the ES. Several lines of evidence support the hypothesis that aldosterone may regulate Na^+^ transport in ES, resulting in endolymph volume regulation. The presence of molecules related to acid/base transport, such as H^+^-ATPase, Na^+^–H^+^ exchanger (NHE), pendrin (SLC26A4), Cl^−^–HCO_3_^−^ exchanger (SLC4A2), and carbonic anhydrase in ES epithelial cells, suggests that acid/base transport is another important one in the ES. Recent basic and clinical studies suggest that aldosterone may be involved in the effect of salt-reduced diet treatment in Meniere’s disease.

## Introduction

The endolymphatic system homeostasis is crucial to maintain the normal function in the inner ear [1]. The stria vascularis in the cochlea, the dark cells in the vestibular organ, and the endolymphatic sac (ES) are mainly involved in the maintenance of homeostasis in the endolymphatic system [1]. Two main homeostatic mechanisms of inner ear fluid regulation have been proposed, i.e., radial and longitudinal endolymph movements [2, 3]. The physiology of the stria vascularis has been clarified on the basis of more studies, whereas the ES physiology is still unknown in many parts because of less research.

One of the pathological findings caused by the disturbance of homeostasis in the endolymphatic system is endolymphatic hydrops, which is known to be the typical pathological finding of Menière’s disease [4, 5]. The obliteration of the endolymphatic sac and endolymphatic duct induces endolymphatic hydrops in experimental animals [6]. Therefore, the ES is assumed to play crucial roles in maintaining the endolymphatic system homeostasis. It is important to know the roles of the ES in the endolymphatic system homeostasis to elucidate the pathogenesis underlying the development of Menière’s disease. Recent research on the ES has revealed the aspects of ion transport in the ES. The present review will outline ion transport and its regulation in the ES on the basis of recent research findings with suggestions for clinical aspects of Menière’s disease from the viewpoint of ion transport in the ES.

## Morphology of ES and classification of ES epithelial cells

The ES is divided into the following three parts on the basis of morphological features: proximal, intermediate, and distal portions [2, 7]. The morphological findings imply that epithelial cells in the intermediate portion may be more functional in ion transport than those in the other portions [2, 8]. The epithelial cells in the intermediate portion have been recently classified electronmicroscopically into two types of cells: mitochondria-rich cells and ribosome-rich cells in the rat [8]. Mitochondria-rich cells have been reported to occupy 20–25% of epithelial cells in the intermediate portion of the rat [8]. Cytoorganelle-rich cells and filament-rich cells reported in the guinea pig [7] and the mouse [9] correspond to mitochondria-rich cells and ribosome-rich cells in the rat, respectively. Mitochondria-rich cells and ribosome-rich cells correspond roughly to light cells and dark cells termed by Lundquist [2], repectively. However, it has been pointed out that both cytoorganelle-rich cells and filament-rich cells in the guinea pig and the mouse [9] and both mitochondria-rich cells and ribosome-rich cells in the rat [8] may be stained lightly or darkly as fixation artifacts by electron microscope. Terms of mitochondria-rich cell and ribosome-rich cell have been widely used [10–12]. Table 1 summarizes the classification of epithelial cells in several species based on the morphological findings [2, 7–9, 13–15].

**Table 1:** The morphological classification of epithelial cells in the intermediate portion of the endolymphatic sac of several species

Species	Cell types	References
Guniea pig	Light cell, dark cell	[2]
	Cytoorganelle-rich cell (type 1) filament-rich cell (type 2)	[7]
Mouse	Light cell, dark cell	[13]
	Cytoorganelle-rich cell (type 1) filament-rich cell (type 2)	[9]
Rat	Light cell, dark cell	[14]
	Mitochondria-rich cell, ribosome-rich cell	[8]
Human	Two types of cells: (1) cells with numerous microvilli and Basal infoldings (2) cells with few microvilli and packed	[15]

## Resting potential and ion concentration in ES endolymph

Endolymph in the ES is quite different in resting potential, ion concentration and pH from that in the other parts of the inner ear, such as the cochlea or vestibular organ (Table 2) [16–27]. It should be stressed that resting potential in the ES is higher than that in endolymph of the vestibular organ, although it is quite lower than that in the cochlea, and that endolymph in the ES has higher Na^+^ and lower K^+^ and Cl^−^ concentrations and lower pH than cochlear and vestibular endolymph.

**Table 2:** Values of resting potential, ion concentration and pH in endolymph and perilymph of the guinea pig

	Resting potential	Ion concentration (mM)					pH	References
		Na^+^	K^+^	Ca^2+^	Cl^−^	HCO_3_^−^		
Endolymph								
Endolymphatic sac	14.7	103.3	11.6	0.47	85	20	6.7	[20, 23, 24, 26, 27]
Saccule	7.3	3	150	0.09	119			[16, 19, 25]
Utricle	4.8	14.3	150	0.13	119			[16, 19, 25]
Semicircular ampulla	3.9	18.4	130.4	0.26				[22, 24]
Cochlea	84	0.23	154.5	0.017	127.8	21.4	7.4	[17, 18, 21, 22, 25]
Perilymph								
Scala tympani	0	144.7	2.7	1.36	124.3	21		[17, 21, 25]
Scala vestibuli	0	141	9		123	18		[17, 21, 25]

Resting potential in the ES named endolymphatic sac potential (ESP) [28, 29] was found by Amano et al. [20]. ESP, which is oxygen-dependent, has the following different properties from resting potentials in cochlear and vestibular endolymph:

1. ESP has no negative potential induced by anoxia [30, 31].
2. ESP shows different responses to several diuretics from resting potentials in cochlear and vestibular endolymph [28, 31–34]. It is less sensitive to loop diuretics, whereas it is more sensitive to canrenoate, an aldosterone antagonist, and acetazolamide, a carbonic anhydrase inhibitor.
3. Catecholamines produce a reversible depression in ESP by β_2_ adrenergic action [35].
4. ESP is mainly composed of an acetazolamide-sensitive part and an isoproterenol-sensitive part [36].

ESP is assumed to have plural origins, one of which may be H^+^-ATPase [37]. The presence of ESP may prompt Na^+^ transport from the ES lumen to the outside although its physiological roles remain to be clarified.

## Molecules related to transport of ion and water in ES epithelial cells

Molecules related to ion transport in epithelial cells in the intermediate portion of the ES are shown in Table 3 [10, 38–60]. The type of cells with most molecules has not been identified except only a few molecules [10, 46]. It should be stressed that Na^+^–K^+^–2Cl^−^ cotransporter 2 (NKCC2) [56, 57, 59, 60] and thiazide-sensitive Na^+^–Cl^−^ cotransporter (NCC) [55, 58], which had been previously recognized to be selectively located in kidney, are present in ES epithelial cells.

**Table 3:** Molecules related to ion transport in epithelial cells of the endolymphatic sac

Molecules	Cell localization	Species	References
Ion channels			
Na^+^ channel (amiloride-sensitive)	Apical membrane	Guinea pig	[38]
Epithelial sodium channel (ENaC)	Apical membrane	Rat, human	[39, 40]
K^+^ channel (outward delayed rectifier)	Basolateral membrane	Guinea pig	[41]
Non-selective cation channel (ATP-activated)	Apical membrane	Guinea pig	[42, 43]
Cystic fibrosis transmembrane conductance regulator (CFTR)	Apical membrane	Rat	[40]
Transient receptor potential vanilloid (TRPV) 4	Apical membrane	Rat, mouse, human	[44, 45, 46]
K^+^ channel (KCNN2, KCNK2, KCNK6, KCNJ14)		Human	[47]
ATPases			
Na^+^–K^+^-ATPase	Basolateral membrane	Guinea pig	[48]
H^+^-ATPase	Apical membrane	Guinea pig, mouse	[49, 50]
Carbonic anhydrase	Membrane, cytoplasm	Guinea pig, mouse, chinchilla	[50, 51, 52, 53]
Ion exchangers			
Cation exchanger: Na^+^–H^+^ exchanger	Apical membrane	Guinea pig, human	[54, 55]
Anion exchangers			
Cl^−^–HCO_3_^−^ exchanger (SLC4A2)	Basolateral membrane	Guinea pig	[49]
Pendrin (SLC26A4)	Apical membrane	Mouse, human	[10, 50, 55]
Cotransporters			
Bumetanide-sensitive Na^+^–K^+^–2Cl^−^ cotransporter 2 (NKCC2)	Apical membrane	Rat, human	[55, 56, 57, 59, 60]
Thiazide-sensitive Na^+^–Cl^−^ cotransporter (NCC, SLC12A3)	Apical membrane	Rat, human	[55, 58]
Na^+^-phosphate cotransporter (SLC34A2)	Apical membrane	Human	[55]
Aquaporins			
AQPs 1–4, 6–9		Rat	[59, 61]
AQPs 1–3		Mouse	[63]
AQPs 1–4		Human	[44, 57, 62]

Several isoforms of aquaporin (AQP) as molecules related to water transport in ES epithelial cells have been reported, as shown in Table 3 [44, 57, 59, 61–63].

## Electrophysiological profile on ion transport in ES epithelial cells

Recent lines of evidence on ion transport in the ES (including cation, anion and acid/base transports) is as follows:

1. ES epithelial cells have resting membrane potential of approximately −60 mV [41].
2. ES endolymph has resting potential of approximately +15 mV [24, 37, 64].
3. ES endolymph has a higher Na^+^ concentration and lower K^+^ and Cl^−^ concentrations. There are active Na^+^ and Cl^−^ outflows from the ES lumen into the outside [20, 24].
4. K^+^ and Na^+^ are permeable ions, but Cl^−^ is a negligible ion in the ES isolated epithelial cells [65].
5. Mitochondria-rich cells in the ES have a higher activity of Na^+^, K^+^-ATPase and a higher Na^+^ permeability [11].
6. ES endolymph has a weak acidity [26], in which H^+^-ATPase may be involved [66].
7. ES epithelial cells have molecules related to acid/base transport, such as H^+^-ATPase [49, 50], Na^+^–H^+^ exchanger (NHE) [54, 55], pendrin (SLC26A4) [10, 50, 55], Cl^−^–HCO_3_^−^ exchanger (SLC4A2) [49], and carbonic anhydrase [50–53].

## Ion transport properties in ES epithelial cells

**Fig. 1:**
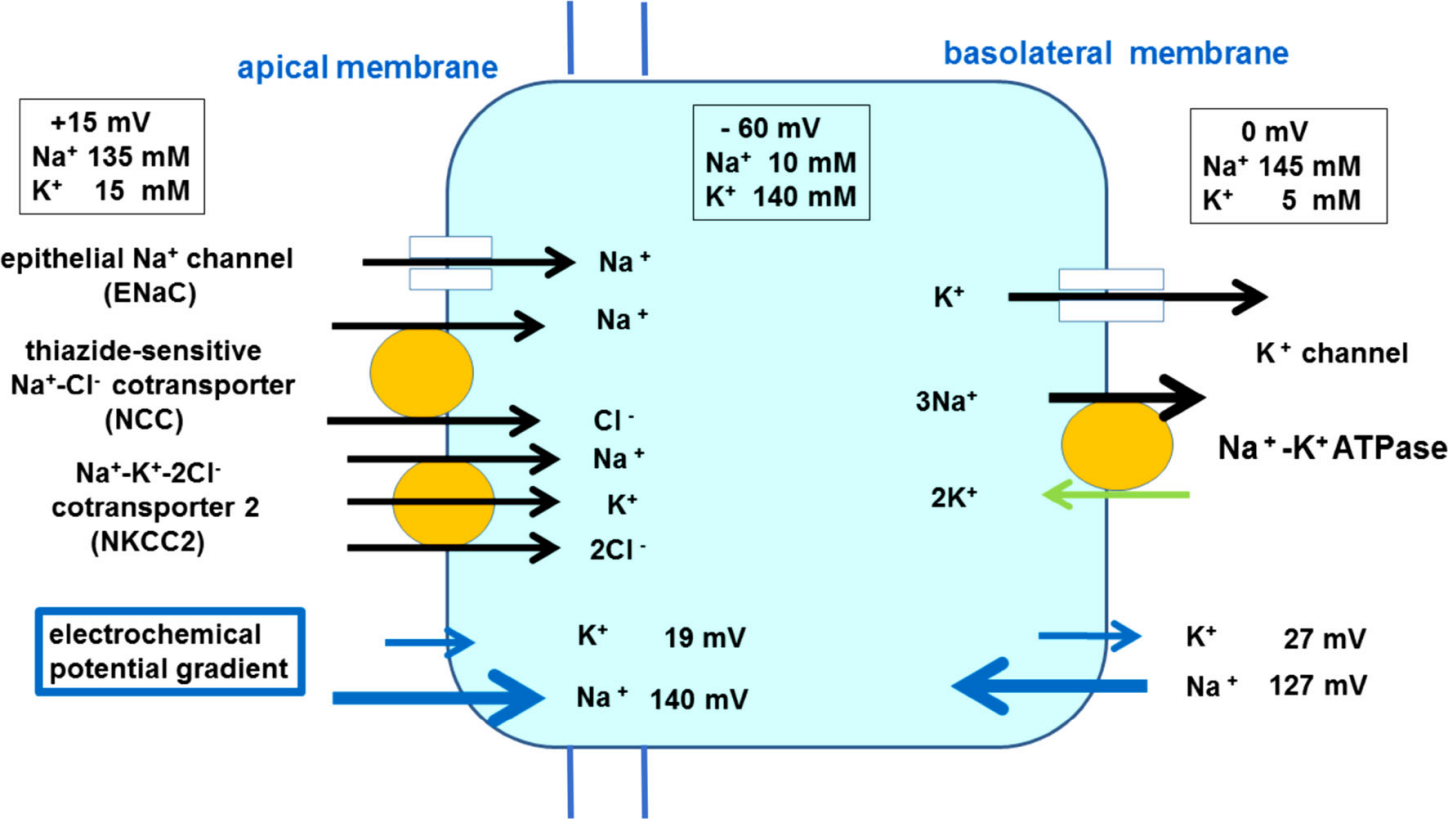
Na^+^ and K^+^ transport model in mitochondria-rich epithelial cells of the endolymphatic sac. Large positive electrochemical gradients for Na^+^ promote Na^+^ inflow into the cell from apical and basolateral membrane. Inflowing Na^+^ is actively transported by Na^+^, K^+^-ATPase with a high activity. Na^+^ absorption is followed by water movement from endolymph to the outside

Our Na^+^ imaging study [11] demonstrates that mitochondria-rich cells in the ES have a higher activity of Na^+^, K^+^-ATPase and a higher Na^+^ permeability, strongly suggesting that molecules related to Na^+^ transport may be densely located in mitochondria-rich cells. Mitochondria-rich cells in ES have characteristic qualities of Na^+^ absorption (Fig. 1). Na^+^ enters the cell across the apical membrane through ion channels and ion transporters driven by an estimated electrochemical driving force of approximately 140 mV. K^+^ enters the cell from endolymph across the apical membrane through the non-selective cation channel driven by an estimated electrochemical driving force of approximately 20 mV and through NKCC2 driven by a higher Na^+^ inflow. Na^+^ is removed across the basolateral membrane by Na^+^, K^+^-ATPase. K^+^ is brought into the cell by the pump, and subsequently diffuses out through K^+^ channel (outward delayed rectifier), which is involved in the maintenance of negative intracellular potential. The model is similar to that found classically in several other Na^+^-absorbing epithelia [67]. Na^+^ transport is a major part of ion transport system in ES.

Molecules related to acid/base transport are H^+^-ATPase, Na^+^–H^+^ exchanger (NHE) and pendrin in the apical membrane, Cl^−^–HCO_3_^−^ exchanger (SLC4A2) in the basolateral membrane, and intracellular and membrane-bound carbonic anhydrase [50]. H^+^-ATPase, pendrin, and carbonic anhydrase have been shown to be localized in the same type of ES epithelial cells [50]. Pendrin has been reported to be present in mitochondria-rich cells [10]. For the maintenance of acidity in ES lumen, the inflow of H^+^ into the lumen is necessary to be larger than the inflow of HCO_3_^−^. NHE in the apical membrane, which is presumed to be active due to a large Na^+^ inflow into the cell, besides H^+^-ATPase may be largely involved in the acidity of ES endolymph. Acid–base transport is another important part of ion transport system in ES.

Molecules related to Cl^−^ transport have been reported to be an ion channel (cystic fibrosis transmembrane conductance regulator, CFTR) in the apical membrane [40], ion cotransporters in the apical membrane (NKCC2 and NCC) [56, 58], and ion exchangers (pendrin in the apical membrane [50], and Cl^−^–HCO_3_^−^ exchanger (SLC4A2) in the basolateral membrane [49]). CFTR has been shown to be co-localized with ENaC [40]. There has been no report on intracellular Cl^−^ concentration of ES epithelial cells. When intracellular Cl^−^ concentration of ES epithelial cells is assumed to be similar to 4 mM in other tissues [68], the electrochemical gradient for Cl^−^ into the cell is calculated to be around 0 mV. Since the results using whole cell patch clamp showed that Cl^−^ current was negligible in the ES isolated epithelial cells [65], it is unlikely that Cl^−^ is a leading ion in ES ion transport system. Cl^−^ is assumed to be transported following Na^+^ transport and acid/base transport.

## Regulation of Na^+^ transport in ES

Several agents, such as vasopressin [44, 69, 70], aldosterone [71], cortisol [72, 73], atrial natriuretic peptide [74], catecholamines [75–77], and ATP [78], have been suggested as the candidates for regulators of ion transport in ES. It has been known that several hormones such as vasopressin and aldosterone regulate Na^+^ transport in other tissues such as the kidney [79, 80, 81]. More lines of evidence supporting aldosterone as a regulator of Na^+^ transport in ES has been accumulated in comparison with other candidates as follows:

1. The presence of mineralocorticoid receptors (MRs) in ES epithelial cells has been shown [71].
2. 11β-hydroxysteroid dehydrogenase type 2 (11β-HSD2), which enables aldosterone selectively to bind to MRs by converting cortisol (corticosterone) into inactive metabolites, has been detected in ES epithelial cells [82]. The presence of 11β-HSD2 is considered essential in aldosterone-target tissues [83]. The absence of 11β-HSD2 has been shown in cochlear and vestibular tissues [84].
3. The presence of ENaC in ES epithelial cell has been shown [40].
4. The presence of NCC in ES epithelial cells has been shown [58]. NCC had been accepted to have a specific localization in distal convoluted tubule of the kidney until NCC was found in ES epithelial cells. NCC is regulated by aldosterone [85].
5. The antagonist of aldosterone, canrenoate, intravenously produced a decreased ESP change with no change in the endocochlear potential, suggesting that aldosterone could act more sensitively on the ES [28, 31].

**Fig. 2:**
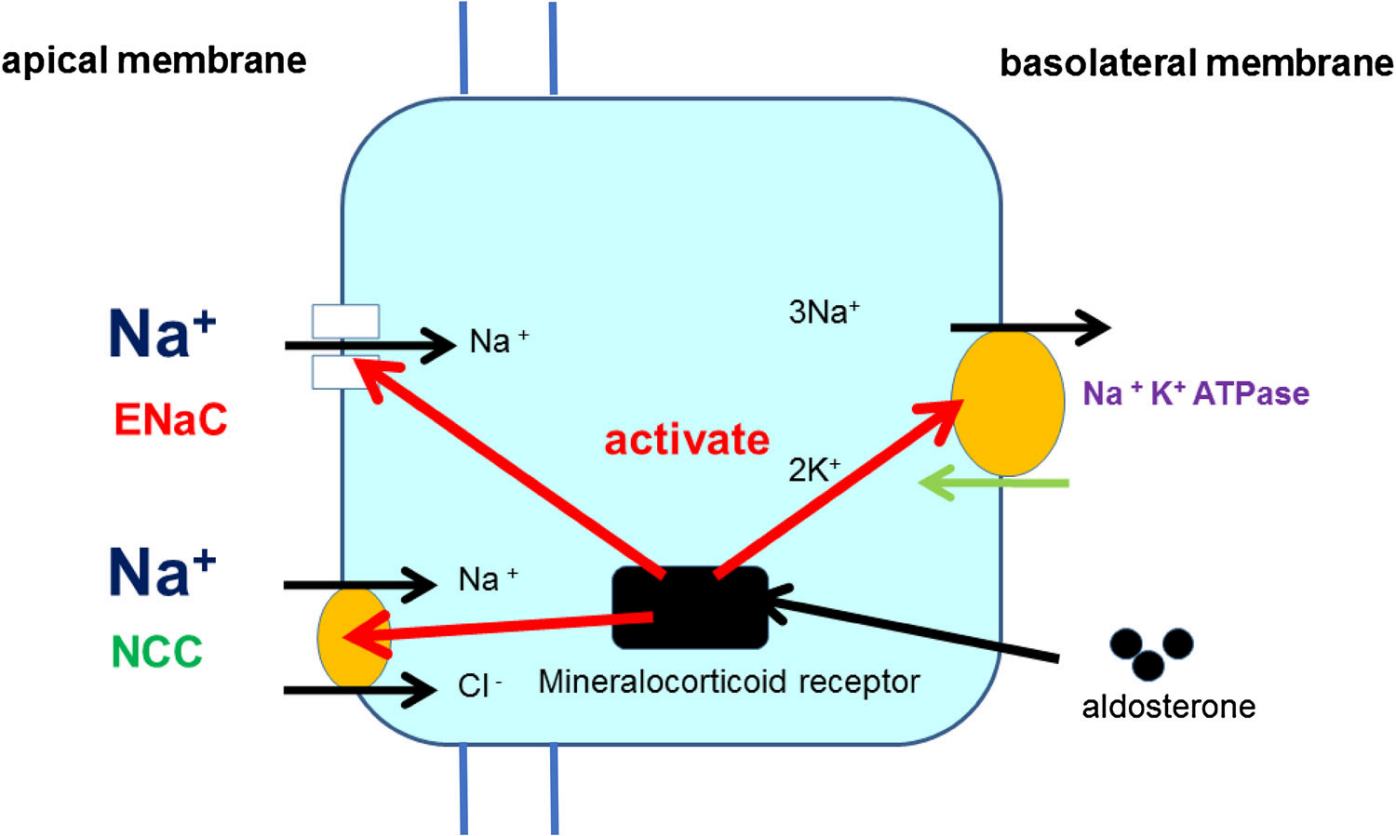
Activation of Na^+^ absorption in ES epithelial cells by aldosterone. Aldosterone activates ENaC and NCC in the apical membrane and Na^+^, K^+^-ATPase in the basolateral membrane through the binding to mineralocorticoid receptors in cytoplasm, resulting in increased Na^+^ absorption in ES epithelial cells

Aldosterone activates Na^+^ transport from endolymph into ES epithelial cells, mainly mitochondria-rich cells in a similar manner to epithelial cells in other aldosterone-target tissues (Fig. 2). Activation of Na^+^ absorption increases water absorption, resulting in increased endolymph absorption.

Several AQP isoforms including AQP2 activated by vasopressin have been detected in the ES epithelial cells, as shown in Table 3. The specific presence of various kinds of AQP isoforms in the ES reinforces more effective water movement accompanying Na^+^ transport, resulting in efficient endolymph absortption in the ES.

## Suggestions for clinical aspects of Meniere’s disease from recent evidence on ion transport in ES

Plasma aldosterone concentration has been reported to be within the normal range in patients with Meniere’s disease [86, 87]. Therefore, the elevation of plasma aldosterone concentration is not considered to be directly involved in the pathogenesis of Meniere’s disease. However, the findings suggesting that aldosterone may be involved in endolymph volume regulation through the regulation of Na^+^ transport in the ES give an experimental support to empirical salt-reduced diet treatment in Meniere’s disease. Our recent study [87] has shown that salt-reduced diet with no administration of thiazide is an effective treatment in Meniere’s disease, and that during 2-year treatment period, salt-reduced diet induced the elevation of plasma aldosterone concentration with no change in other hormones, such as vasopressin, cortisol, and brain natriuretic peptide in patients with Meniere’s disease as the elevation of plasma aldosterone concentration has been reported in patients with hypertension [88]. The presence of NCC in the ES besides the kidney [55, 58] may propose a necessity to reconsider the indication of thiazide in Meniere’s disease.

The presence of vasopressin-AQP-2 system in ES epithelia may play important roles in endolymph volume regulation [44, 61], suggesting that vasopressin-AQP-2 system in the ES would be involved in the development of Meniere’s disease [70, 89, 90].

The findings that catecholamines increased the hydrostatic pressure of cochlear and vestibular endolymph [76, 77] probably through β adrenergic action on the ES give a basic support to the clinical empirical finding that the stress often worsens the symptoms in patients with Meniere’s disease. Results that the degree of an increase in endolymphatic hydrostatic pressure induced by β agonist was significantly larger in the pars inferior than that in the pars superior [77] may give any suggestions in considering the clinical course in Meniere’s disease.
